# Near-Wall Migration Dynamics of Erythrocytes *in Vivo*: Effects of Cell Deformability and Arteriolar Bifurcation

**DOI:** 10.3389/fphys.2017.00963

**Published:** 2017-11-29

**Authors:** Bumseok Namgung, Yan Cheng Ng, Hwa Liang Leo, Joseph M. Rifkind, Sangho Kim

**Affiliations:** ^1^Department of Biomedical Engineering, National University of Singapore, Singapore, Singapore; ^2^Biomedical Institute for Global Health Research and Technology, National University of Singapore, Singapore, Singapore; ^3^NUS Graduate School for Integrative Sciences and Engineering, National University of Singapore, Singapore, Singapore; ^4^Department of Anesthesiology and Critical Care Medicine, Johns Hopkins Medicine, Baltimore, MD, United States

**Keywords:** hemodynamics, microcirculation, bifurcation flow, RBC deformability, RBC margination

## Abstract

Red blood cell (RBC) deformability has a significant impact on microcirculation by affecting cell dynamics. Despite previous studies that have demonstrated the margination of rigid cells and particles *in vitro*, little information is available on the *in vivo* margination of deformability-impaired RBCs under physiological flow and hematocrit conditions. Thus, in this study, we examined how the deformability-dependent, RBC migration alters the cell distribution under physiological conditions, particularly in arteriolar network flows. The hardened RBCs (*h*RBCs) were found to preferentially flow near the vessel walls of small arterioles (diameter = 47.1–93.3 μm). The majority of the *h*RBCs (63%) were marginated within the range of 0.7*R*-0.9*R* (*R*: radial position normalized by vessel radius), indicating that the *h*RBCs preferentially accumulated near the vessel walls. The laterally marginated *h*RBCs maintained their lateral positions near the walls while traversing downstream with attenuated radial dispersion. In addition, the immediate displacement of RBCs while traversing a bifurcation also contributes to the near-wall accumulation of *h*RBCs. The notable difference in the inward migration between the marginated *n*RBCs and *h*RBCs after bifurcations further supports the potential role of bifurcations in the accumulation of *h*RBCs near the walls.

## Introduction

At physiological levels of hematocrit (35–45%), red blood cells (RBCs) are continuously subjected to inter-cellular collisions in the microvascular network. Accordingly, alterations in RBC aggregation and deformability are expected to lead to a substantial modification in the distribution of RBCs over the microvascular network. In previous studies, a pathological elevation of RBC aggregation has been shown to result in the formation of a thicker cell-free layer (CFL) near the vessel wall (Ong et al., 2011), which can promote a plasma-skimming effect (Fung, 1973) resulting in a reduction in microvascular perfusion (Namgung et al., 2015).

In addition, RBC deformability is a key physical determinant in the distribution of RBCs in the microcirculation. Normal RBCs are highly deformable and thus able to travel through capillaries even narrower than their diameter. A pathological decrease in RBC deformability, however, leads to an elevation in the peripheral vascular resistance and consequently a decrease in the tissue perfusion (Simchon et al., 1987). Moreover, RBC deformability is an important factor determining the sequestration of the aged RBCs in the spleen (MacDonald et al., 1987; Mebius and Kraal, 2005) as well as affecting the shear-dependent behavior of blood viscosity (Chien, 1987). The clinical significance of RBC deformability has long been highlighted (Weed, 1970; Chien, 1987; Mokken et al., 1992) in many diseases such as sepsis (Condon et al., 2003), malaria infected blood (Dondorp et al., 2000, 2002), and in prolonged storage of blood (Haradin et al., 1969; Stuart and Nash, 1990).

Despite the clinical significance of RBC deformability, there exists limited information of its potential effects on cell migration in the microcirculation. A previous *in vitro* study, Hou et al. (2010) reported the outward lateral migration (margination) of less deformable malaria-infected RBCs toward the channel walls. It was further highlighted that an elevation in hematocrit promoted the cell–cell interaction between the normal and less-deformable RBCs, which leads to an enhanced displacement of the malaria-infected cells toward the walls (Hou et al., 2010). In addition, a recent *in vivo* study, Lee et al. (2013) showed that ~70% of rigid particles (diameter = 1 μm) infused into mice preferentially accumulated near the vessel walls (0.8*R*−1.0*R, R*: normalized radial position by vessel radius) in small vessels (diameter = 15–30 μm). It was also demonstrated that less deformable (stiff) particles were increasingly localized near the walls, whereas deformable (floppy) particles had enhanced hydrodynamic migration away from the walls resulting in their accumulation near the center of the vessel (Kumar and Graham, 2012a,b).

Such redistribution of RBCs would lead to local hematocrit changes. This could consequently influence the plasma-skimming effect and hematocrit partitioning in bifurcations (Schmid-Schonbein et al., 1980; Pries et al., 1989; Enden and Popel, 1994; Barber et al., 2008; Li et al., 2012). In a previous *in vitro* study (Shevkoplyas et al., 2006), RBC perfusion in a microchannel network was found to be highly sensitive to RBC deformability. It was demonstrated that the attenuated plasma skimming effect in *h*RBCs eventually led to blockages of capillary channels (ID = 5 μm) and also a more heterogeneous distribution of RBCs in the microchannel network. Importantly, this could in turn alter the microvascular oxygen and metabolite distributions as well as the effective viscosity of blood in the microvessels (Pries et al., 1996).

Consecutive multiple branching of the microvasculature is an essential morphological feature, which allows extensive perfusion to all peripheral tissue regions. This *in vivo* bifurcation have a major impact on flow patterns through the microcirculation. The impact of the arteriolar network is indicated by the finding that the travel distance required for less deformable RBCs to show a distinct margination in a straight vessel (Hou et al., 2010) has been reported to be one (or two) order of magnitude greater than an inter-bifurcation distance in the arteriolar network (Pries et al., 1989; Kiani et al., 1993; Ong et al., 2012). In this study, we examined how the diverging structure of the arteriolar networks contribute to the disparity in the migration rates of deformable and rigid RBCs toward the flow center. The radial distribution of chemically-hardened RBCs (*h*RBCs) was quantified *in vivo* after their infusion into the rat circulatory system. The contribution of bifurcations was examined using *in vitro* microchannel studies to further examine the suspension characteristics of the *h*RBCs in the presence of bifurcations.

## Methods

### Animal preparation

All animal handling procedures were in accordance with *National University of Singapore Institutional Animal Care and Use Committee Guidelines and Ethics on Animal Experimentation* (approved protocol no. R15-0225). A total of six male rats (Sprague-Dawley) weighing 183 ± 27 g were used for the *in vivo* study. The animals were initially anesthetized with the ketamine (37.5 mg/mL) and xylazine (5 mg/mL) cocktail (2 mL/kg) through intraperitoneal injection. The surgery was performed on a heating pad while maintaining the body temperature at 37°C. The animal was tracheotomized to assist breathing. The jugular vein was catheterized for the administration of additional anesthetic and dextran solutions. The femoral artery was catheterized for the withdrawal of blood sample and real-time pressure monitoring (Biopac TSD 104A, Goleta, USA). All catheters were heparinized with saline (30 IU/mL) solution to prevent blood clotting. The rat cremaster muscle was exteriorized to visualize arteriolar network flows. After the surgical exposure of the muscle, warm Plasma-Lyte A (35°C, pH 7.4; Baxter, USA) was continuously applied to the muscle to keep it moist. Nerves and blood supply were ensured to be remained intact. The muscle was then stretched and secured on a Plexiglas platform for the clear visualization of the desired blood vessels. The platform was fitted with two heating elements to maintain the muscle temperature at 35°C during the experiment. The muscle was then irrigated with the Plasma-Lyte A before covering it with a polyvinyl film (Saran, S. C. Johnson & Son, Singapore). At the end of the experiment, the animal was euthanized with an overdose of pentobarbital sodium.

### Microscopic systems and image acquisition

After the surgical preparation, the rat was placed on the microscopic stage and left to stabilize over a period of ~10 min. The arterial cannulation was connected to a physiological data-acquisition system (MP 100 System, BIOPAC Systems, USA) for continuous arterial pressure monitoring during the experiment. A straight arteriole [Inner diameter (ID) < 100 μm] located at least two vessel diameters away from the upstream bifurcation was selected for tracking labeled cells. All video recordings were taken with the criteria of a stable flow, clear image focus and good image contrast. An intravital microscope (BX51, Olympus, Japan) was used with a 40X water-immersion objective (LUMPlanFL 40xW, Olympus, Japan). Fluorescent images were acquired for 60 s, with a sCMOS fluorescent camera (Pco.edge, PCO AG, Kelheim, Germany) that provides the resolution of 512 by 512 (pixel by pixel) at 200 frame/s. All image analysis was performed with a custom-built MATLAB script (Mathworks, Natick, MA, USA).

### RBC labeling and hardening

Whole blood from a donor rat was withdrawn and transferred into a heparinized tube. The blood was then centrifuged at 2,500 g for 10 min (Sigma 2-6, Goettingen, Germany). The buffy coat and plasma were gently removed after the first centrifugation, and the remaining RBCs were washed three times more with 1X Phosphate Buffer Saline (PBS, pH 7.4, Life Technologies, CA, USA). Subsequently, the normal RBCs (*n*RBCs) were labeled with PKH-67 (PKH67 Green Fluorescent Cell Linker, Sigma-Aldrich, USA) at a final concentration of 2 μM. The labeling of RBCs with PKH-67 has been widely used in previous studies for reliable cell viability (Deplaine et al., 2011; Progatzky et al., 2013). The labeled RBCs were washed three times and hardened RBCs (*h*RBCs) were prepared by additionally incubating the cells in glutaraldehyde (GA) solution (Grade I, 25% in H_2_O, Sigma-Aldrich, USA) at 1.0 mM for 30 min at room temperature. After the incubation, the cell mixture was washed three times with PBS and resuspended in saline (NaCl 0.9%, B. Braun, Melsungen, Germany) at the same hematocrit as that found in the recipient rat to maintain the total blood volume and the systemic hematocrit in the circulatory system after the exchange-transfusion. The GA-treated *h*RBCs were stored at 4°C before the RBC exchange. The deformability difference between the *n*RBCs and *h*RBCs was verified using a commercially available ektacytometer (RheoScan-D, RheoMeditech, Korea) that provides the elongation index (EI) defined by the ellipsoid diffraction pattern.

### Adjustment of RBC aggregation and exchange of *h*RBCs

To ascertain the hemorheological relevance to humans, the degree of rat blood aggregation was adjusted to levels seen in normal human blood. This was achieved by infusing a total of 200 mg/kg of Dextran 500 (Avg. MW = 450–550 kDa, Pharmacosmos A/S, Denmark) dissolved in saline into rats over the course of 1–2 min to obtain plasma-dextran concentrations of ~0.63% in the rat blood (Ong et al., 2011). The level of RBC aggregation (M-index) was determined with an optical aggregometer (Myrenne Aggregometer MA-1, Myrenne GmbH, Roetgen, Germany) at the stasis mode (M0). The dextran concentration used in this study induced aggregating conditions to that found in normal human blood (*M* = 12–16) (Ong et al., 2011; Namgung and Kim, 2014).

Whole blood in the recipient rat was exchange-transfused with the *h*RBCs. The target volume of *h*RBCs for the exchange was ~10% of the total RBC population by assuming that the total blood volume of the rat is 5.5% of its body weight (Bishop et al., 2001). The target volume of whole blood was first withdrawn from the femoral artery before the infusion of the *h*RBCs. Immediately after the withdrawal, the *h*RBCs were infused at ~100 μL/min via the jugular vein (Cabrales et al., 2005).

A blood sample (<0.1 mL) was withdrawn from the femoral artery for hematocrit and aggregation measurements. Hematocrit was determined with a microhematocrit centrifuge (Sigma 1–14 Microcentrifuge, Sigma, Germany). All the blood sample measurements were repeated before and 15-min after the dextran infusion and *h*RBC exchange.

### Determination of *h*RBCs radial position

**Figure 1:**
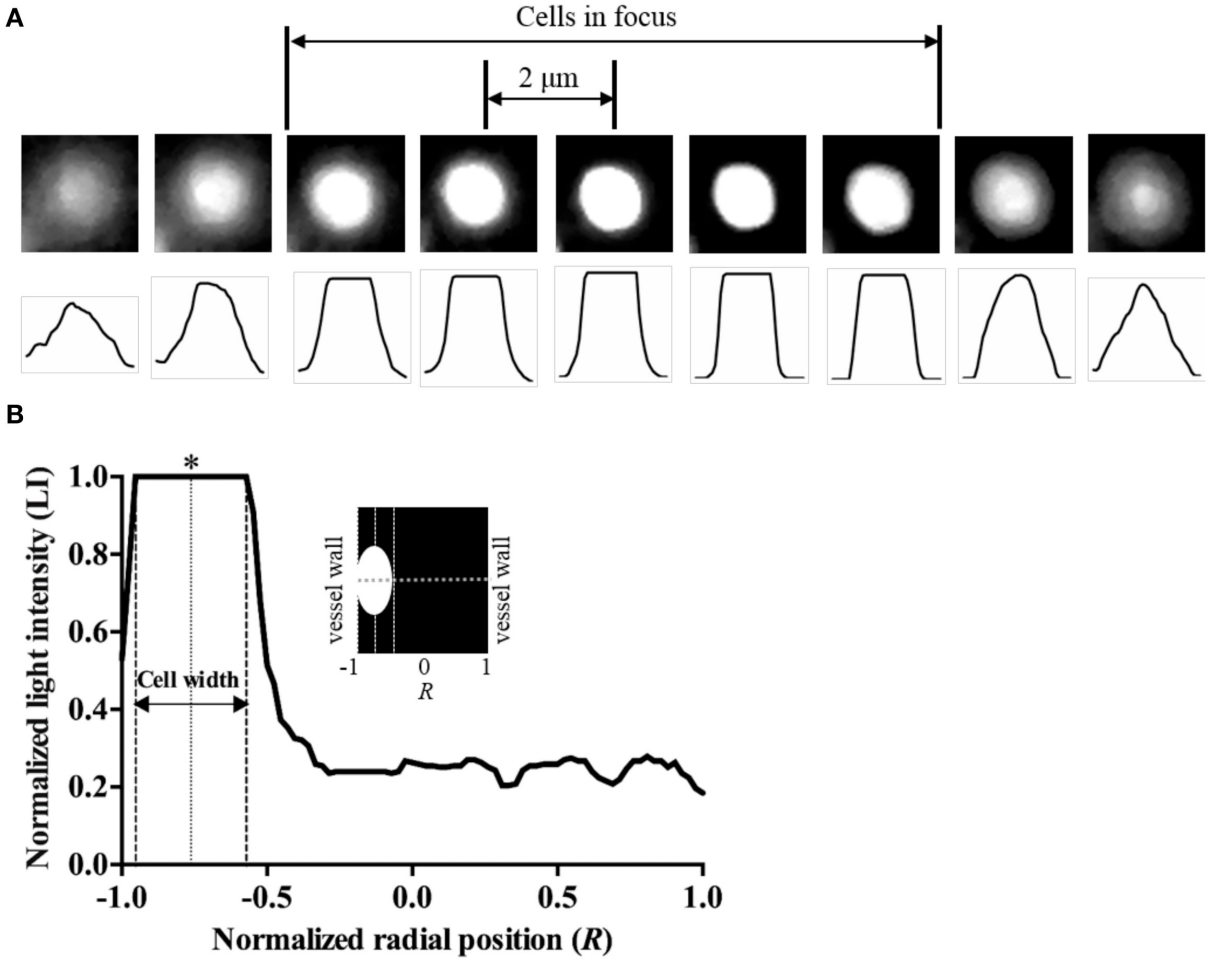
Determination of radial position of cells. **(A)** Microscopic images of a labeled *h*RBC in stasis condition and corresponding line intensity profiles with 2-μm vertical shifts of the microscope stage. **(B)** Typical light intensity profile across the vessel with a RBC flowing near the vessel wall. Radial position of the cell was determined at the center of the cell width (marked as ^*^).

To determine the radial position of the *h*RBCs in the equatorial plane of the vessel, a previously reported approach (Bishop et al., 2001) was adopted in this study (Figure 1A). Random scattered noise in the image was minimized with a median filter for better detection of the fluorescent-labeled *h*RBCs. The labeled cells in an 8-μm section (cells in focus) showed a clear and sharp transition of intensity profile from dark to white. Based on this criterion, we differentiated between cells in and out of focus. The radial position of the cells (*R*) was defined at the center of the cell width normalized by the vessel radius (Figure 1B), where *R* ranges from 0 (flow center) to 1 (vessel wall). Since the radial position of the cells was determined at the center of cells (Figure 1B), it is noted that the radial position of cells adjacent to the wall cannot be 1.0*R* in our results.

### *In vitro* microchannel study

*In vitro* microchannels were fabricated with polydimethylsiloxane (PDMS, Dow Corning, MI) by the standard soft lithography and replica molding techniques. The detailed design of the microchannel is depicted in Supplementary Figure S1. The labeled target RBCs (*n*RBC or *h*RBC) were suspended in the PBS-dextran solution (~0.63%) to simulate the *in vivo* aggregating condition, and then flowed in the microchannel at 1% hematocrit. To initialize the lateral position of the target RBCs at the outer wall of the main branch, a sheath RBC fluid (*n*RBCs at 40% hematocrit in the PBS-dextran solution) was introduced from another inlet. The lateral positions (*L*) of the target RBCs were analyzed at every 5*D* before (from −50*D*_m_ to 0*D*_m_) and after the bifurcation [from 0*D*_d_ to 25*D*_d_) (*D*: travel distance along microchannel normalized by the channel width of the main (m) or daughter (d) channel]. The baselines of each branch segment (0*D*_m_ or 0*D*_d_) were determined at the end of the filleted corners (see Supplementary Figure S1). The inlet flow rates for the target RBCs and the sheath fluid were controlled using a syringe pump (KDS210, KD Scientific, Holliston, MA) at 1 and 2.6 μL/min, respectively.

### Statistical analysis and data presentation

A statistical software package (Prism 6.0, GraphPad) was used for all statistical analyses. Two-tailed unpaired *t*-tests were performed to determine the statistical difference between two groups. All physiological and rheological values were represented as means ± SD. The analysis of covariance (ANCOVA) was performed to determine the difference between two slopes is significant. *p* < 0.05 was considered statistically significant.

## Results

### Systemic parameters and RBC deformability

**Figure 2:**
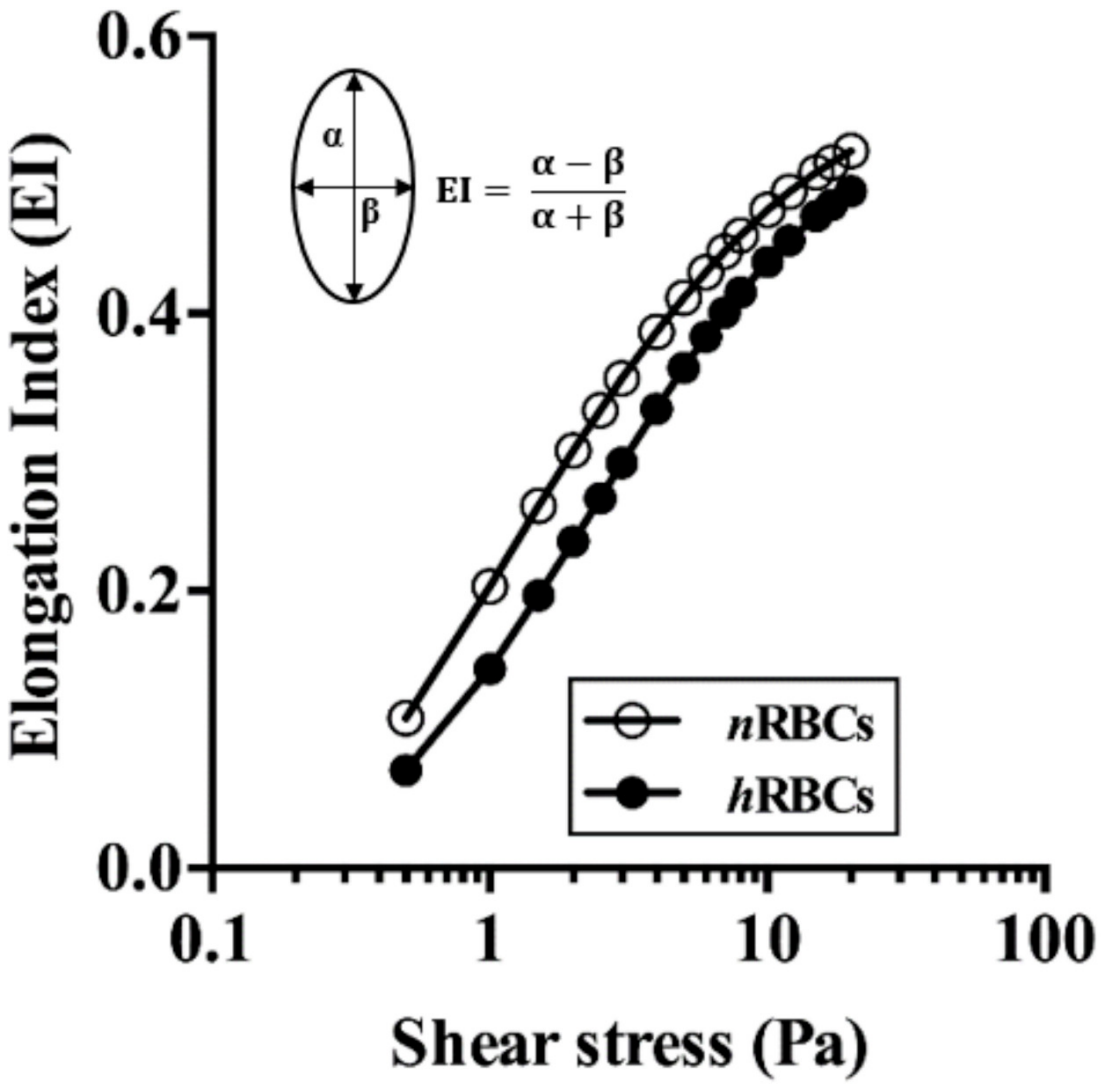
Deformability of *n*RBCs and *h*RBCs. EI is defined by major (α) and minor (β) axes of ellipsoid diffraction pattern from the ekatacytometer. Standard deviation was too small to be noticed (*n* = 4).

No statistical difference was found in the systemic parameters (MAP, ID, PSR, Hct and M-value) before and after the exchange of *h*RBCs as presented in Table 1. The *h*RBCs were consistently less deformable than *n*RBCs over a wide range of shear stresses (τ = 0.5–20 Pa) as shown in Figure 2. Thus, the GA treatment used in this study resulted in a noticeable reduction in EI by 35% at τ = 0.5 Pa and 5.6% at τ = 20 Pa.

**Table 1:** Systemic parameters.

***h*RBCs exchange**	**MAP (mmHg)**	**ID (μm)**	**PSR (s^−1^)**	**Hct (%)**	***M*-value**
Before	94 ± 17	76 ± 16	63 ± 17	42 ± 1.0	13.8 ± 2.5
After	93 ± 19	75 ± 17	52 ± 9	41 ± 1.0	13.2 ± 1.8

*Values are in mean ± SD. MAP, mean arterial pressure; ID, inner diameter of vessel; PSR, pseudoshear rate (mean velocity / ID); Hct, hematocrit; M-value, aggregation index*.

### Radial distribution and migration of *h*RBCs *in vivo*

**Figure 3:**
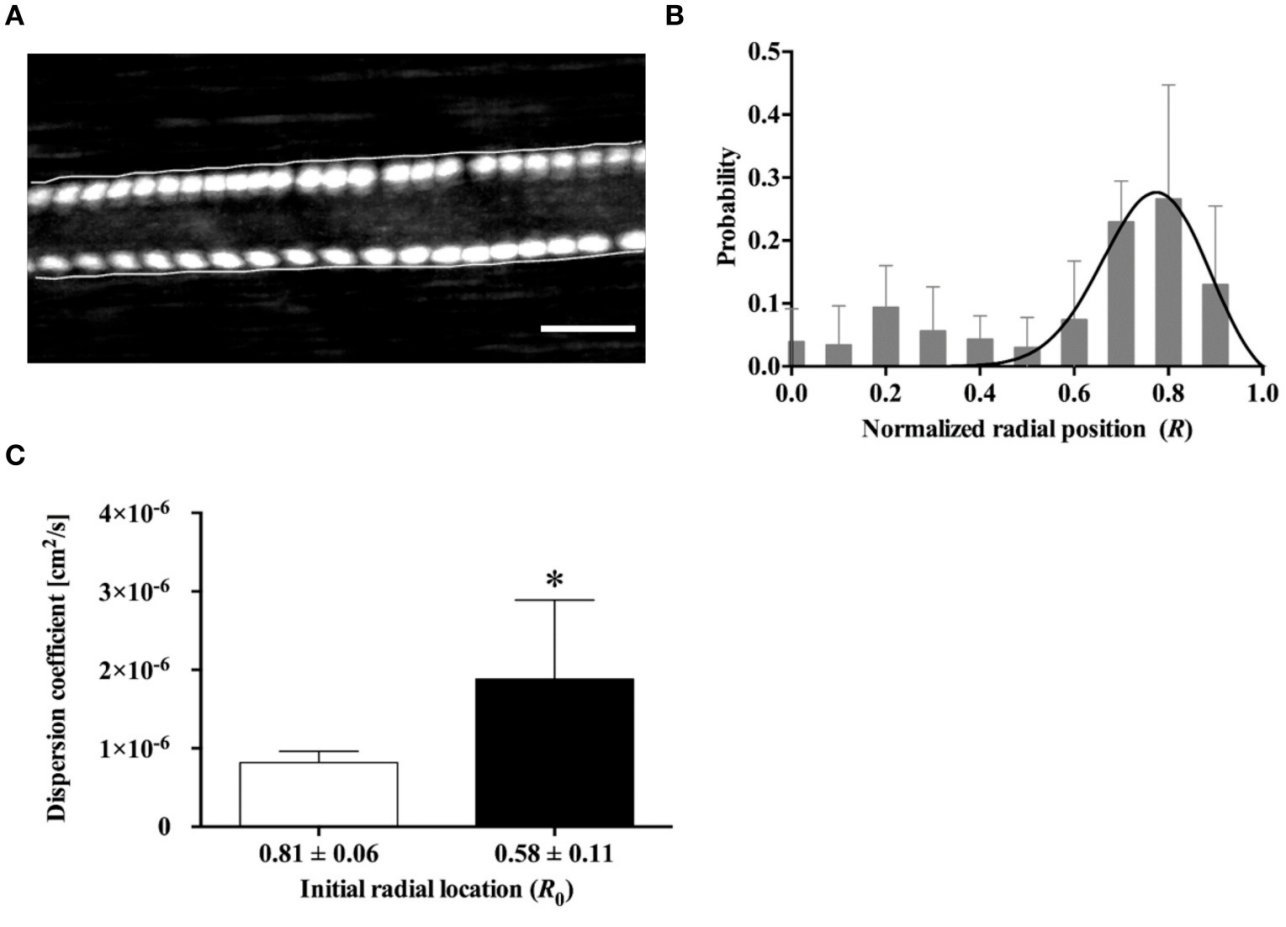
Radial distribution of *h*RBCs *in vivo*. **(A)** Fluorescent image of two labeled *h*RBCs flowing near the vessel walls in an arteriole (ID = 55.1 ± 2.9 μm, averaged along longitudinal direction). The overlaid cell trajectory image was obtained by continuously taking the maximum intensity of the consecutive images of cell flow (cumulative maximum intensity image). Scale bar = 50 μm. **(B)** Probability distribution for the radial position of *h*RBCs (A total of 112 cells were analyzed from six different vessels). A solid line represent a hyperbolic regression fit for experimental data (*y* = *a*·tanh(1 – *x*)·sech^*b*^ (*x – c*) where *a* = 1.443, *b* = 62.23, *c* = 0.8431; *R*^2^ = 0.4). **(C)** Dispersion coefficient of *h*RBCs at *R*_0_ = 0.81 and *R*_0_ = 0.58 (*n* = 7); ^*^p < 0.05.

Figure 3A shows a typical example of *h*RBC flow in an arteriole. The two *h*RBCs maintained their radial positions near the opposite walls of the vessel during the flow. We analyzed a total of 112 *h*RBCs for their radial locations (Figure 3B). The non-linear regression curve clearly shows that ~28% (at the maximum peak) of the *h*RBCs were flowing at the radial position of 0.8*R*. Furthermore, the majority of the *h*RBCs (63%) were marginated within the range of 0.7*R*−0.9*R*, indicating that the *h*RBCs preferentially accumulated near the vessel walls.

In addition, the radial dispersion of the *h*RBCs was examined based on the initial locations (*R*_0_) of the cells by tracking their trajectories along the vessel. To provide a quantitative description on the dispersion of the cells, a dispersion coefficient (*D*_RBC_) of the *h*RBCs was determined: *D*_RBC_ = Δ*R*^2^ / 2Δ*t*, where Δ*R* represents a radial displacement during a given time interval (Δ*t*) (Bishop et al., 2002). It was observed that *h*RBCs initially located near the vessel wall (*R*_0_ = 0.81) showed a significantly (*p* < 0.05) lower *D*_RBC_ than those located away from the wall (*R*_0_ = 0.58) (Figure 3C).

### Immediate margination of *h*RBCs at a bifurcation *in vivo*

**Figure 4:**
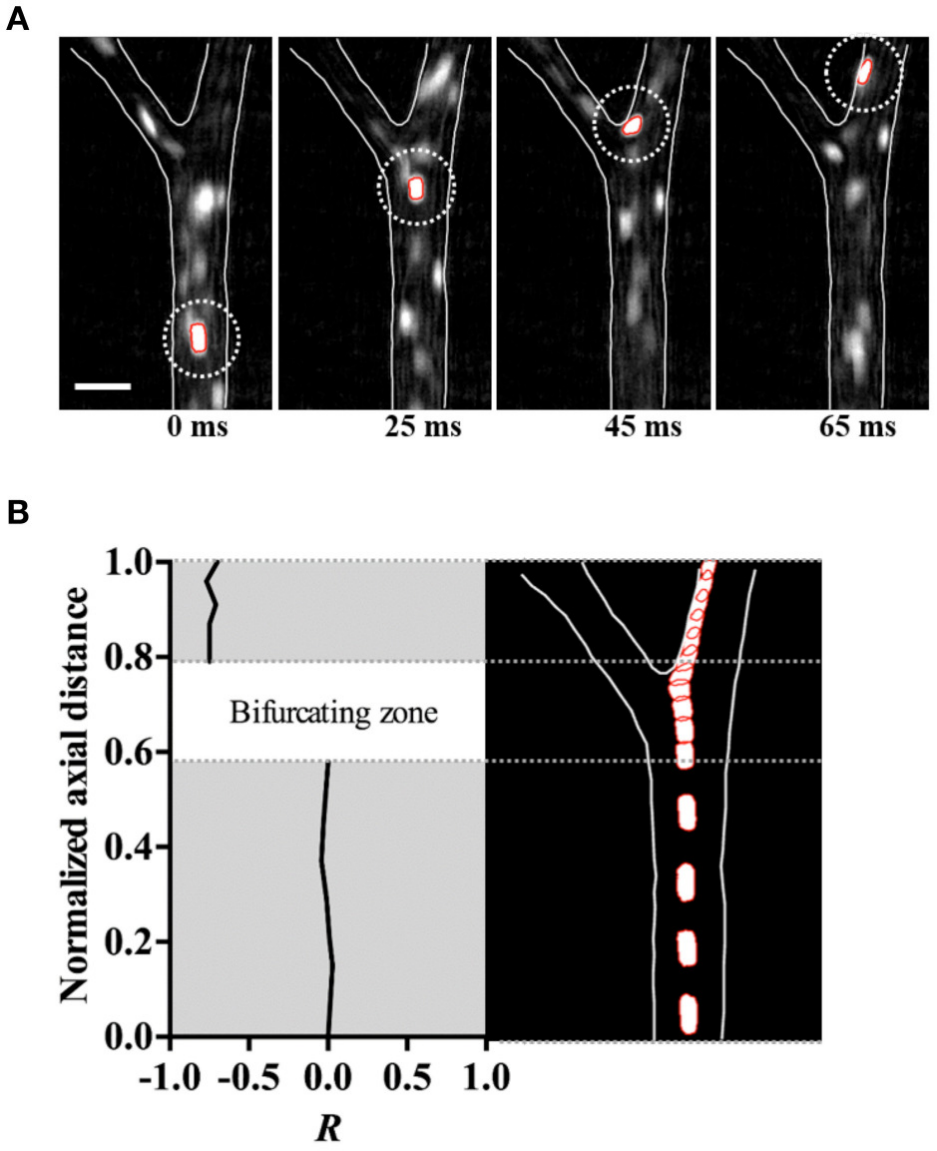
Immediate margination of *h*RBC. **(A)** Consecutive *in vivo* images of *h*RBC (white dotted circle) flowing over 65 ms in an arteriole (ID = 50.2 ± 1.5 μm). Scale bar = 50 μm. **(B)** Normalized radial position (*R*) (left) and trajectory (right) of *h*RBC in **(A)**.

The trajectory of the *h*RBCs was additionally examined along the upstream section of an arteriolar bifurcation, particularly at the inner vessel wall near the bifurcating point. We found that the *h*RBCs exhibited an abrupt shift in their radial positions immediately after the bifurcation (Figure 4A). Specifically, the *h*RBC, initially located near the center (0*R*) of the parent vessel and aligned with the bifurcating point, seemed to be relocated at the wall (0.75*R*) of the downstream daughter vessel (Figure 4B). This is expected since the cells near the center streamline of the parent vessel eventually coincide with the apex of the bifurcation, thus leading to their repositioning near the vessel wall.

### Disparity in inward migration of *n*RBCs and *h*RBCs at a bifurcation

**Figure 5:**
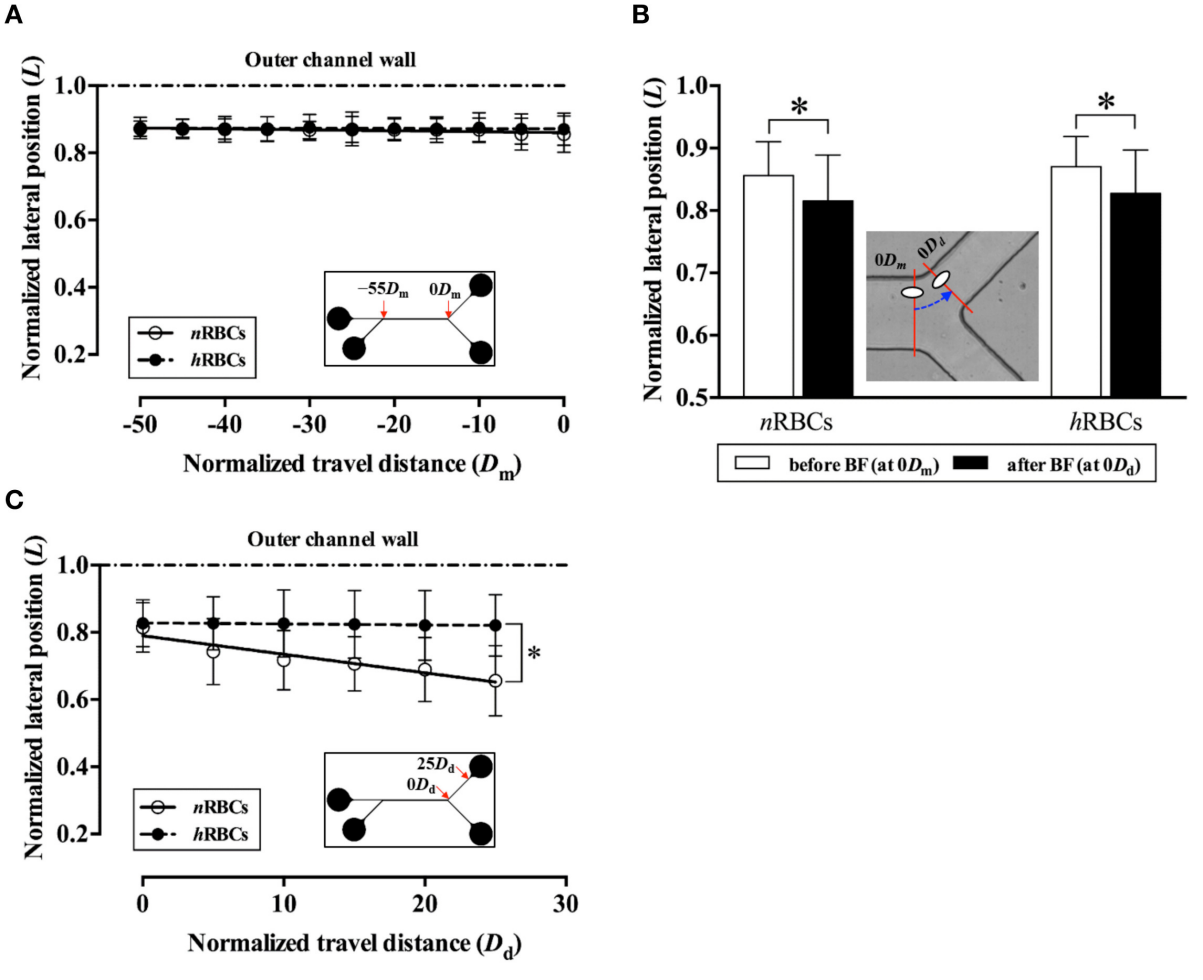
Difference in migration dynamics between *n*RBCs and *h*RBCs across the bifurcation (BF) in the *in vitro* system. **(A)** Normalized lateral position (*L*) of the target RBCs in the main channel before the bifurcation. The lateral position (*L*) and travel distance (*D*_m_) of the target RBCs were normalized by the half width (*w*/2) and the full width (*w*) of the channel, respectively. Each line (solid and dashed) represents a linear regression fit of the data. (*y* = −2.8 × 10^−4^*x* + 0.86; *R*^2^ = 0.61 for *n*RBCs, *y* = −0.44 × 10^−4^*x* + 0.87; *R*^2^ = 0.17 for *h*RBCs). **(B)** Normalized lateral position (*L*) of the target RBCs at 0*D*_m_ and 0*D*_d_ across the bifurcation. The dashed line with an arrow indicates the transit of RBC (^*^p < 0.0001). **(C)** Normalized lateral position (*L*) of the target RBCs in the daughter channel after the bifurcation. Each line (solid and dashed) represents a linear regression fit of the data. (*y* = −5.51 × 10^−3^*x* + 0.79; *R*^2^ = 0.90 for *n*RBCs, *y* = −0.30 × 10^−3^*x* + 0.83; *R*^2^ = 0.91 for *h*RBCs; ^*^p < 0.0001; significant difference between two slopes). A total of 300 cells (150 *n*RBCs and 150 *h*RBCs) were analyzed for each data point.

For comparison of migration tendencies between *n*RBCs and *h*RBC, an *in vitro* microchannel study was performed to overcome the technical limitations of our *in vivo* study including the limited field of view for tracking target cells and the limited tracking time due to photo bleaching. Particularly, to further elucidate the flow dynamics of the marginated RBCs (*n*RBCs and *h*RBCs) at a bifurcation, a Y-bifurcation microchannel was used to examine the difference in the inward migration of the laterally displaced RBCs (Supplementary Figure S1). No discernible difference was observed between the migration of *n*RBCs and *h*RBCs from −50*D*_m_ to 0 *D*_m_ in the main channel (Figure 5A). Moreover, both the laterally displaced *n*RBCs and *h*RBCs did not exhibit any statistically significant inward migration despite the long travel distance in the straight region of the microchannel.

Interestingly, as the target RBCs traverse through the bifurcation from 0*D*_m_ to 0*D*_d_ (Figure 5B), they exhibited a slight, but significant (*p* < 0.0001) inward migration toward the flow center regardless of the cell rigidity (*n*RBC: 0.86*L* → 0.82*L, h*RBCs: 0.87*L* → 0.83*L*). However, the difference in the lateral position between *n*RBCs and *h*RBCs at 0*D*_d_ progressively increased along the daughter branch (Figure 5C). In particular, the lateral position of the *n*RBCs (0.82*L*) was ~20% farther away from the outer wall than the *h*RBCs (0.66*L*) at 25*D*_d_. Accordingly, the inward migration tendency of *n*RBCs was significantly greater than that of the *h*RBCs (*p* < 0.0001) after the bifurcation. Similar to our *in vivo* observations in this study (Figure 3B), the *h*RBCs persistently retained their lateral positions near the wall. It is also noteworthy that the lateral position of the *h*RBCs (0.82*L*) at 25*D*_d_ falls within the range of the radial positions of the *h*RBCs observed *in vivo* (0.8*R*).

## Discussion

In this study, rat RBCs were chemically hardened with glutaraldehyde (GA) solution (1.0 mM). GA is a non-specific protein cross-linker that induces cross-linking in all the components of the cell (cytosol, cytoskeleton, transmembrane) (Forsyth et al., 2010; Sosa et al., 2013), thus resulting in an overall increase in the RBC rigidity similar to a single-component solid particle. In the present study, the deformability of GA-treated RBCs (*h*RBCs) was consistently lower than that of the *n*RBCs based on the EI measured over the range of shear stresses (0.5-20 Pa). Previously, it was reported that the averaged wall shear stress in an arteriole (ID = 29.5–67.1 μm) of the rat cremaster muscle ranged from ~0.3 (MAP = 47 ± 5.5 mmHg) to 7.5 Pa (108 ± 4.7 mmHg) (Namgung et al., 2011). Another study reported that the arteriolar wall shear stress (ID = 160–220 μm) ranged from 1.0 to 4.5 Pa under physiological flow conditions (Bakker et al., 2003). Thus, under such physiological ranges of shear stresses, the difference in RBC deformability found between the *h*RBCs and *n*RBCs (5.6–35% at τ = 25–0.5 Pa in Figure 2) could lead to alterations in the suspension characteristics, which in turn result in the preferential near-wall accumulation of *h*RBCs. However, it is of note that the EI-based measurement of the RBC deformability only indicates the ability of the cell to elongate. RBCs are in general subjected to various types of deformations (folding, bending, and elongating) while traversing the microvasculature. Therefore, the GA-induced decrease in the RBC deformability possibly results in a different suspension behavior as compared to that of the pathologically hardened RBCs in different diseases.

It has been known that in arteriolar flows, the formation of CFL near the vessel wall is due to the shear-induced inward migration of *n*RBCs toward the flow center and their accumulation in the core region. Previous computational studies (Pranay et al., 2012; Hariprasad and Secomb, 2014) demonstrated that the inward migration of cells (or elastic capsules) arises from fluid mechanical interactions between the cells and the vessel walls. Particularly for RBCs, their inward migration is enhanced by the tank-treading motion of their flexible membrane. In contrast, the outward migration is elicited mainly from shear-induced dispersions due to interactions between the cells (Pranay et al., 2012; Hariprasad and Secomb, 2014). In this study, the near-wall margination (outward migration) of *h*RBCs shown in Figure 3B could be attributed in part to the inward migration of *n*RBCs, which is similar to that reported for the margination of leukocytes (Freund, 2007; Munn and Dupin, 2008). The preferential margination of leukocytes to the vessel walls at a physiological level of hematocrit is mainly due to the continuous hydrodynamic collisions with neighboring flexible *n*RBCs (Schmid-Schönbein et al., 1980; Goldsmith and Spain, 1984). In addition, the relatively faster inward migration of the *n*RBCs also partly contributed to such a margination phenomenon (Goldsmith and Spain, 1984). The rotation of the deformable *n*RBC membrane around its center of mass (tank-treading motion) promotes the axial migration, which subsequently displaces it away from the vessel walls (Dupire et al., 2012). In contrast, *h*RBCs displayed an enhanced tumbling motion (Forsyth et al., 2010) resulting in a diminished tank–treading motion. This attenuation in the tank-treading motion of the *h*RBCs is expected to limit their inward migration toward the flow center.

The marginated *h*RBCs (*R*_0_ → 1) tended to maintain their radial positions with reduced radial dispersions (Figures 3B,C). In contrast, the significantly higher dispersion of *h*RBCs near the RBC core region (*R*_0_ → 0.5) implied that the cell–cell interaction was dominant in the region where the cells were more concentrated. Similarly, a previous *in vivo* study Lee et al. (2013) reported that the dispersion of rigid 1-μm particles in the blood stream was greatly reduced near the walls relative to the RBC core region. Although the marginated *h*RBCs remained in contact with the boundary of the RBC core while flowing near the walls, they were unable to penetrate into the boundary. This suggests that the *h*RBCs were in an equilibrium position (Munn and Dupin, 2008). In addition, the attenuated deformability-dependent wall lift force experienced by the *h*RBCs potentially further contribute to the decreased radial dispersion near the wall. Accordingly, it was previously reported that deformable RBCs were affected by the lift force ranging from 31 to 155 pN at wall shear stresses between 0.2 to 1 Pa, whereas undeformed spherical leukocytes experienced zero lift force (Abkarian and Viallat, 2008).

It is important to note that the outward migration of *h*RBCs in a straight vessel requires a long travel distance to complete their near-wall margination induced by the hydrodynamic interaction with neighboring cells. A previous study Jain and Munn (2009) showed that the number of marginated leukocytes increased almost linearly with the length (up to 5 mm) of a rectangular microchannel with widths of 50 and 75 μm. A separate *in vitro* study, Hou et al. (2010) demonstrated the separation of malaria-infected RBCs (more rigid than *n*RBCs) based on their near-wall margination, which was achieved with an extreme channel ratio of 1: 2,000 (width: length). However, such extremely long and straight vessels are not observable in the microvasculature. The mean inter-bifurcation distance was reported to be approximately 5 to 33 vessel-diameter in arterioles (ID = 4–104 μm) of the rat cremaster muscle (Ong et al., 2012), rat mesenteric muscle (Pries et al., 1989) and hamster cremaster muscle (Kiani et al., 1993). Therefore, based on the previously proposed mechanism for the cell margination, the complete near-wall margination of *h*RBCs is not observed within the short inter-bifurcation distance in arteriolar network flows. A previous computational study, Müller et al. (2016) highlighted that rigid particles generally exhibit greater margination than soft particles. This could be explained by the increased lift force with correspondingly larger deformations that drives deformable particles away from the wall. Therefore, the elevation in rigidity of RBCs under pathological conditions would further increase their margination rate.

The immediate displacement of the RBCs at the inner vessel wall along the bifurcating point (Figure 4) seemed to contribute to the near-wall margination of *h*RBCs observed *in vivo* (Figure 3B). This phenomenon is likely amplified by the multiple bifurcations present in the arteriolar networks, thus resulting in the enhanced accumulation of *h*RBCs near the vessel walls. Due to the limited field of view of our microscopic system, it was unfeasible to perform a quantitative analysis on the radial position of *h*RBCs in a series of bifurcations *in vivo*. However, it is expected that the marginated *h*RBCs maintain their radial positions near the walls due to the reduced dispersion after the bifurcation (Figure 3C). This is in good agreement with a previous numerical study, Takeishi et al. (2014) which showed that the margination of leukocytes was sustained by the continuous passing motion of the RBCs in the RBC core (Takeishi et al., 2014).

The RBCs flowing at the outer channel wall exhibited a sudden inward shift by ~5% in their lateral locations (*n*RBC: 0.86*L* → 0.82*L, h*RBCs: 0.87*L* → 0.83*L*) immediately after the bifurcation (Figure 5B). This is likely due to a sudden local reduction in hematocrit adjacent to the outer wall which is induced by the asymmetric velocity profile developed at the bifurcation (Sherwood et al., 2014), resulting in thicker CFL formation near walls. The enhanced CFL formation at the outer wall (Ong and Kim, 2013) allows cells to migrate inward with minimal collisions with the neighboring cells. This was subsequently verified in our supplementary *in vitro* experiments using diluted RBC suspensions (Hct = 0.2%). The target RBCs in the diluted suspension showed a larger extent of sudden displacement away from the outer wall after the bifurcation (Supplementary Figure S2) as compared to the target RBCs in the concentrated suspension (Figure 5B). This strongly supports that the local reduction in hematocrit and the CFL formation near the outer wall at the bifurcation provide the marginal space for cells to exhibit an accelerated inward migration. Consequently, the *n*RBCs were able to cross the streamlines toward the flow center more effectively with a larger extent of deformation and tank-treading motions than the *h*RBCs. This becomes more apparent at physiological levels of hematocrit since the cell–cell interactions effectively attenuate the inward migration of *h*RBCs but not the *n*RBCs (Figure 5C).

**Figure 6:**
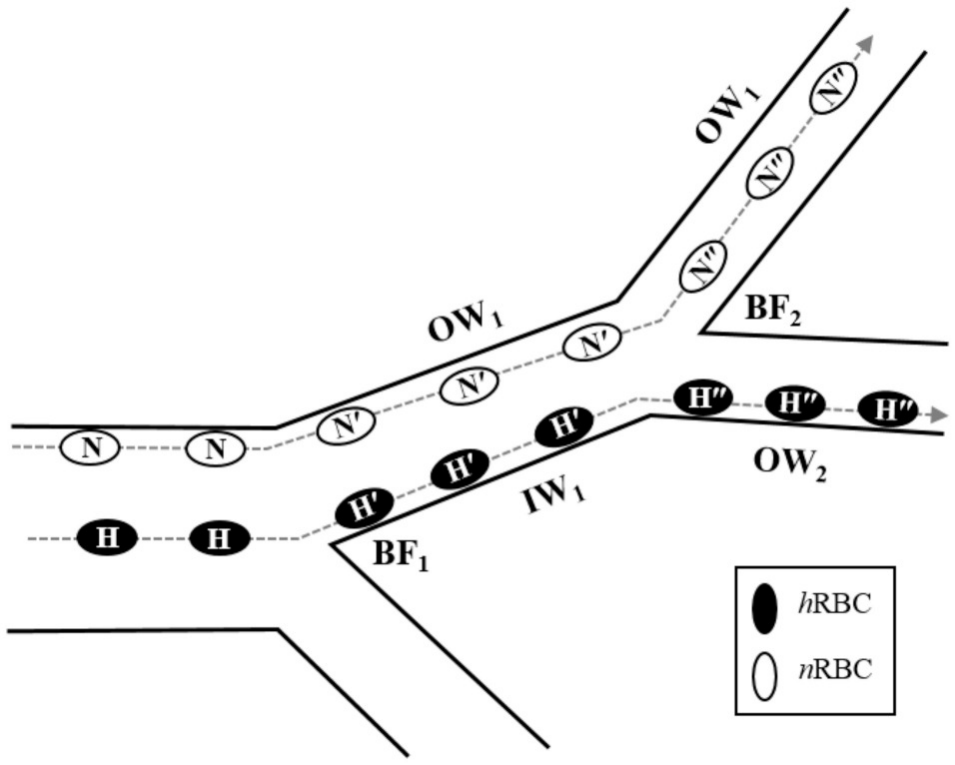
Potential role of bifurcation in radial migration of *n*RBCs (N) and *h*RBCs (H). The dashed lines with arrows indicated the trajectories of each RBC. N′ and H′ represent the *n*RBCs and *h*RBCs after the 1st bifurcating point (BF_1_) while N″ and H″ represent the *n*RBCs and *h*RBCs after the 2nd bifurcating point (BF_2_), respectively. OW and IW stand for the outer and inner walls, respectively.

The cumulative contribution of bifurcations to the accumulation of *h*RBCs near the vessel walls *in vivo* is depicted in Figure 6. In the blood flow, *h*RBCs located near the flow center are relocated to the inner wall (H → H′) after a bifurcating point (BF_1_). Due to their diminished inward migration, *h*RBCs maintain their lateral positions near the inner wall farther downstream until the next bifurcating point (BF_2_), where the inner wall in the upstream vessel becomes the outer wall in the downstream vessel (IW_1_ → OW_2_). As the *h*RBCs traverse through BF_2_ (H′ → H″), they continue to remain their marginated positions along the walls. In contrast, laterally displaced *n*RBCs are able to migrate progressively toward the flow center (N → N′ → N″) in the downstream vessels after a series of bifurcations. This is due to the enhanced inward migration of *n*RBCs as well as the sudden inward shift toward the flow center at the bifurcations as observed in the present study. The disparity in the migration between *n*RBC and *h*RBC across the bifurcations consequentially leads to the selective accumulation of *h*RBCs near the vessel walls.

The near-wall accumulation of *h*RBCs could contribute to the decrease in the mean CFL width (Zhang et al., 2009). Under moderate RBC aggregation and a discharged hematocrit of ~38%, the mean normalized CFL width was predicted to decrease from 0.269 to 0.152 due to the 20-time increase in RBC rigidity. Accordingly, the increase in membrane rigidity prevented large deformation and reduced the cell–cell contact area, thus resulting in the formation of a less dense RBC core and thinner CFL. Although a direct comparison between the previous study and our findings is limited due to the stark differences in the magnitude of cell rigidity and volume concentration of *h*RBCs used, the decreasing trend in the CFL width due to the hardening of RBCs would still be expected. Importantly, the reduction in the CFL width could lead to a myriad of physiological influences including flow resistance (Maeda et al., 1996; Zhang et al., 2009), oxygen delivery and nutrients exchange (Tateishi et al., 2001), as well as wall shear stress-dependent nitric oxide (NO) bioavailability (Vaughn et al., 1998; Ng et al., 2015).

## Conclusion

This study shows that a change in the overall RBC deformability significantly alters the distribution of the RBCs in arterioles. The laterally marginated *h*RBCs tend to maintain their lateral positions near the walls while traversing downstream with attenuated radial dispersion. Moreover, the immediate displacement of RBCs while traversing a bifurcation also contributes to the *in vivo* near-wall accumulation of *h*RBCs. The notable difference in the inward migration between the marginated *n*RBCs and *h*RBCs after the bifurcation further supports the contribution of bifurcations to the accumulation of *h*RBCs near the walls.

## Author contributions

BN: Involved in study design, conducted *in vivo* experiments, analyzed the data and prepared the manuscript; YN: Conducted *in vitro* experiments, analyzed the data and revised the manuscript; HL: Verified results and revised the manuscript; JR: Verified results and revised the manuscript; SK: Designed and coordinated research, verified results and revised the manuscript.

### Conflict of interest statement

The authors declare that the research was conducted in the absence of any commercial or financial relationships that could be construed as a potential conflict of interest.
