# Supplementary material for: Near-Wall Migration Dynamics of Erythrocytes in Vivo: Effects of Cell Deformability and Arteriolar Bifurcation
Source: Front Physiol. 2017 Nov 29;8:963. doi: 10.3389/fphys.2017.00963 (PMC5712576; doi:10.3389/fphys.2017.00963)
Supplement: Supplementary file 1 [file Table1.docx]

**SUPPLEMENTARY INFORMATION**

**Near-wall migration dynamics of erythrocytes *in vivo*: Effects of cell deformability and arteriolar bifurcation**

Bumseok Namgung^1,2^, Yan Cheng Ng^1,3^, Hwa Liang Leo^1^, Joseph Rifkind^4^ and Sangho Kim^1,2,3,*^

^1^Department of Biomedical Engineering, National University of Singapore, Singapore, Singapore

^2^Biomedical Institute for Global Health Research and Technology, National University of Singapore, Singapore, Singapore

^3^NUS Graduate School for Integrative Sciences and Engineering, National University of Singapore, Singapore, Singapore

^4^Department of Anesthesiology and Critical Care Medicine, Johns Hopkins Medicine, Baltimore, Maryland, USA

*Corresponding author

Sangho Kim, PhD

Department of Biomedical Engineering, National University of Singapore

4 Engineering Drive 3, Block E4 #04-08

Singapore 117583

Phone: 65-6516 6713

Fax: 65-6872 3069

Email: bieks@nus.edu.sg

**
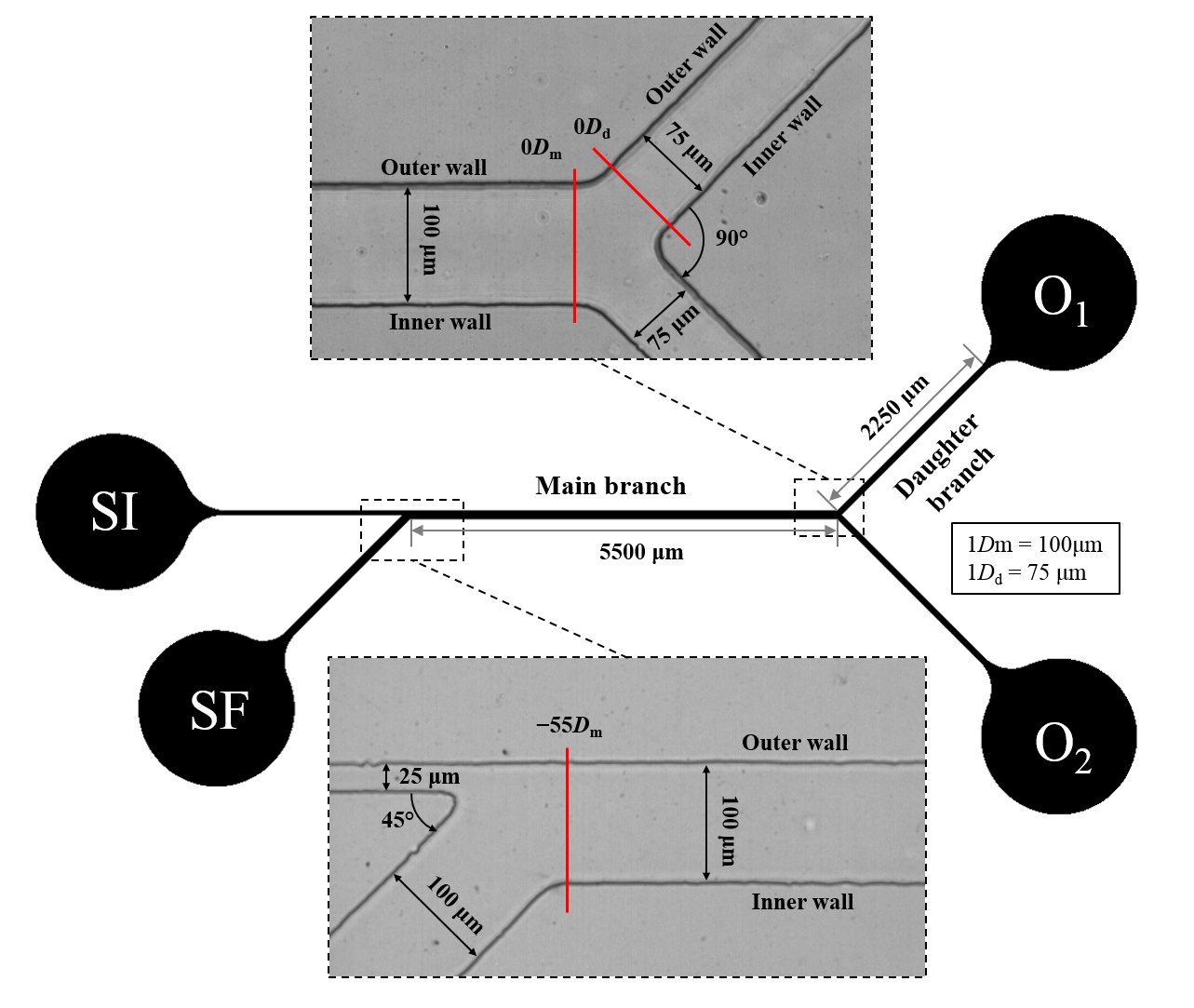
**

**Supplementary Figure S1. Detailed design of the microchannel.**  Solid lines (red) across the channel indicate the locations of the analysis lines for the lateral position of target RBCs. Analysis was performed along the outer walls from −50*D*_m_ to 0*D*_m_ in the main channel and 0*D*_d_ to 25*D*_d_ in the daughter branch. *D*_m_ and *D*_d_ indicate the travel distance normalized by the width of main and daughter branches, respectively (1*D*_m_ = 100 μm, 1*D*_d_ = 75 μm). Flow direction was from left to right. (SI: sample inlet, SF: sheath fluid inlet, O_1_ and O_2_: outlets 1 and 2)

**
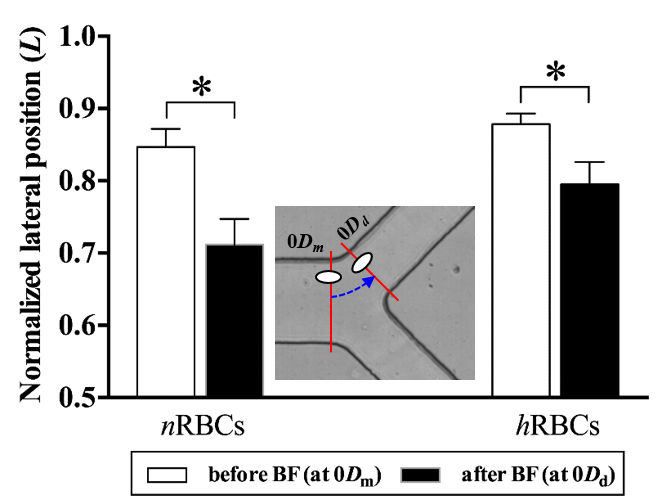
**

**Supplementary Figure S2.** **Normalized lateral position (*L*) of target RBCs in a diluted suspension at 0*D*_m_ and 0*D*_d_ across the bifurcation (BF).** The RBCs flowing near the outer channel wall exhibited a sudden inward shift by ~17% in their lateral locations (*n*RBC: 0.85*L* → 0.71*L*, *h*RBCs: 0.88*L* → 0.80*L*) after the bifurcation. The target RBC sample was prepared at the concentration of 0.2%. The PBS-dextran solution was used as the sheath fluid as described in *Methods*. A total of 50 cells were analyzed for each group. The dashed line with an arrow indicates the transit of RBC. (* *p* < 0.0001)
